# When and Where of Auditory Spatial Processing in Cortex: A Novel Approach Using Electrotomography

**DOI:** 10.1371/journal.pone.0025146

**Published:** 2011-09-19

**Authors:** Jörg Lewald, Stephan Getzmann

**Affiliations:** 1 Department of Cognitive Psychology, Faculty of Psychology, Ruhr University Bochum, Bochum, Germany; 2 Leibniz Research Centre for Working Environment and Human Factors, Dortmund, Germany; Hotchkiss Brain Institute, University of Calgary, Canada

## Abstract

The modulation of brain activity as a function of auditory location was investigated using electro-encephalography in combination with standardized low-resolution brain electromagnetic tomography. Auditory stimuli were presented at various positions under anechoic conditions in free-field space, thus providing the complete set of natural spatial cues. Variation of electrical activity in cortical areas depending on sound location was analyzed by contrasts between sound locations at the time of the N1 and P2 responses of the auditory evoked potential. A clear-cut double dissociation with respect to the cortical locations and the points in time was found, indicating spatial processing *(1)* in the primary auditory cortex and posterodorsal auditory cortical pathway at the time of the N1, and *(2)* in the anteroventral pathway regions about 100 ms later at the time of the P2. Thus, it seems as if both auditory pathways are involved in spatial analysis but at different points in time. It is possible that the late processing in the anteroventral auditory network reflected the sharing of this region by analysis of object-feature information and spectral localization cues or even the integration of spatial and non-spatial sound features.

## Introduction

Spatial hearing is an important feature in human perception. Thus, several efforts have been made to identify areas of the human cerebral cortex that are specialized in the localization of sound sources. However, this topic is still a matter of debate. The most influential hypothesis, initially based on investigations in nonhuman primates, has been derived from the visual cortical system. In analogy to the original model of visual processing in cortex by Ungerleider and Mishkin [1], it was proposed that auditory cortical areas are organized in two segregated pathways. *(1)* An anteroventral (“what”) pathway – primarily processing non-spatial information on spectrotemporal characteristics of sound which connects the primary auditory cortex to anterior temporal lobe and inferior frontal lobe. *(2)* A posterodorsal (“where”) pathway – preferentially processing information on sound location which connects the primary auditory cortex to posterior temporal lobe, posterior parietal lobe, and finally dorsolateral frontal lobe [2]–[8]. In order to investigate whether this hypothesis applies also to the human cortex, several neuroimaging studies have focused on the question of auditory spatial versus object-feature processing by contrasting tasks of localization and spectral analysis (e.g., [9]–[14]). Arnott et al. [15] performed a meta-analysis of 36 functional magnetic resonance imaging (fMRI) and positron-emission tomography (PET) studies, in which subjects completed either “spatial” (e.g., discrimination of sound location) or “non-spatial” auditory tasks (e.g., pitch discrimination). These authors argued that more “spatial” than “non-spatial” studies reported activation in the inferior parietal lobule (IPL) and in the region around the superior frontal sulcus (SFS); activation in the anterior regions of the temporal lobe (aTL) and the inferior frontal gyrus (IFG) was obtained in more “non-spatial” than “spatial” studies and activation in posterior regions of the temporal lobe was observed in both types of studies equally. Even though the general idea of such a functional segregation of posterodorsal and anteroventral auditory pathways had thus received support from the majority of neuroimaging studies, some revisions of the original dual-pathway model are recently under discussion, in which the ventral stream is assigned to perceptual auditory functions while dorsal areas are rather concerned with the preparation of action in response to auditory stimuli [16], [17]. This would largely parallel the (present generally accepted) revision of the visual dual-stream model [18], [19].

The present study aimed to reveal the “spatial” auditory areas in human cortex using a novel combination of methodological approaches that differed from all previous work on this topic. Firstly, in order to investigate the pattern of cortical processing of sound location electro-encephalography (EEG) recordings of auditory-evoked potentials (AEPs) were employed in combination with standardized low-resolution brain electromagnetic tomography (sLORETA; [20]), offering maximum temporal resolution in the millisecond range and acceptable spatial resolution of functional tomographic imaging for 3D localization of intracranial electrical activity (approximately 5 mm). Secondly, unlike almost all related previous imaging studies (as an exception, see [21]), we used stimulation in the free sound field instead of headphones, thus providing the complete set of undistorted localization cues to the auditory system under natural hearing conditions. Thirdly, by refining the approach of preceding functional magnetic resonance imaging (fMRI) studies [22], [23], we focused on the separate analysis of activations evoked by different sound directions and the computation of contrasts between these conditions. Since our goal was to reveal cortical areas involved in spatial processing (in absolute, not relative, terms), we deliberately refrained from contrasting “spatial” and “non-spatial” stimuli, such as performed in the majority of earlier imaging studies (for review, see [15]). Furthermore, no specific task was used (passive listening), as we were specifically interested in genuine sensory rather than sensorimotor processes.

Beyond the localization of areas processing auditory information, the primary focus of this study was to clarify whether different areas are active and different aspects of auditory spatial information (side or eccentric position of the stimulus) are analyzed at specific points in time. We concentrated our analyses on the commonly measured “N1-P2” complex, consisting of the first negative deflection (N1 [24]) and the second positive deflection (P2 [25]) of the AEP. The N1 and P2 are generally considered to be functionally distinct responses, originating from different neural generators: the P2 may reflect a more complex evaluation of stimulus features than the N1, and the P2 sources are located in more anterior areas than N1 sources (e.g., [26]). Thus, it is reasonable to assume that these two components could reflect a dissociation (with respect to locations of neural generators and points in time) of different aspects of auditory spatial processing in human cortex.

In the context of the current discussion on the functional separation of the two auditory pathways two alternative hypotheses could be tested. On the one hand, if the posterodorsal pathway would primarily represent a “spatial” processing (“where”) stream and the anteroventral pathway would be primarily a “non-spatial” processing (“what”) stream, our expectation was that the former, rather than the latter, would show space-specific variation in auditory evoked electrical activity. On the other hand, if the significance of both these pathways is related to functions requiring the supply of auditory spatial information, we expected similar spatial sensitivity in both posterodorsal and anteroventral auditory areas.

## Materials and Methods

### Subjects

Eighteen healthy right-handed subjects (9 female, mean age 25.6 years; range 20–42 years) with normal hearing (by self-report) participated in the experiments. All subjects gave their written informed consent to participate in the study, which was approved by the Ethical Committee of the Medical Faculty of the Ruhr University Bochum. This study conformed to the Code of Ethics of the World Medical Association (Declaration of Helsinki), printed in the British Medical Journal (18 July 1964). Each participant completed one experimental session. Subjects were paid for their participation.

### Apparatus

The listener sat on a vertically adjustable chair in an dimly lighted, anechoic room (4.4 m wide×5.4 m long×2.1 m high), which was insulated by 40 cm (height)×40 cm (depth)×15 cm (width at base) fiberglass wedges on each of the six sides. A suspended mat of steel wires served as floor. The ambient background noise sound-pressure level was below 20 dB(A). The position of the listener's head was held constant by a custom-made chin rest. An array of 91 broad-band loudspeakers (SC 5.9, Visaton, Haan, Germany) was mounted in front of the listener with a distance of 1.5 m from the centre of the head. The loudspeakers were arranged at ear level in the horizontal plane ranging from −90° (left) to 90° (right) in steps of 2°, with the centre loudspeaker at 0°. All loudspeakers were selected on the basis of similar efficiency and frequency response curves. In this experiment, auditory stimuli were presented from sixteen loudspeakers, located at 80°, 70°, 60°, 50°; 40°, 30°, 20°, and 10° to the left and right of the subject's median plane. A red light-emitting diode (LED; diameter 3 mm, luminance 0.025 mcd) located immediately below the central loudspeaker served as a visual fixation target.

### Stimuli

The auditory stimulus was generated digitally using CoolEdit 2000 (Syntrillium Software Co., Phoenix, AZ, USA). It consisted of continuous, band-pass-filtered (lower and upper cut-off frequencies 250 Hz and 20 kHz, respectively), 100-Hz sine-waveform modulated (modulation depth 12%; starting phase 0°) white noise. As we used frozen-noise stimuli, five samples of the stimulus (differing by the waveform of the noise) were generated offline to minimize habituation effects. Stimuli were converted to analogue form via a PC-controlled, 16-bit soundcard (Audigy 2NX, Creative Labs, Singapore) at a sampling rate of 96 kHz and were presented at a sound-pressure level of 50 dB(A). Sound pressure level was measured at the subject's head position, using a sound level meter with a ½-inch free-field measuring microphone (Type 2226, Brüel & Kjær, Nærum, Denmark). We used a moderate level in order to minimize potential effects of noise annoyance with the passive listening paradigm. Each stimulus had a duration of 150 ms (rise/decay times 20 ms).

### Procedure

Unlike the majority of related studies, but as in a previous fMRI study, we employed a methodological approach in which subjects listened passively to the sound stimuli rather than performing any active task of localization. This was deliberately done in order to exclude contamination of the electrotomography imaging data by activations resulting from the subject's responses and to minimize the effects of attention and/or arousal, as our focus was on genuinely sensory processes rather than sensorimotor or higher-order cognitive functions [23], [26], [27]. As suggested by recent single-unit recordings in the monkey primary auditory cortex, responses observed during passive listening may provide a valid representation of neuronal spatial tuning properties [28].

Prior to the experiment, listeners were informed that they would hear sounds from various locations and that they only had to listen passively to the sounds. Furthermore, they were instructed to fixate on the central LED without directing their eyes to the source of the sound. Besides minimizing eye-movement artifacts on auditory ERPs (see below), this instruction aimed to avoid effects of eccentric eye position on processing of sound location, as has previously been described (cf., e.g., [29], [30]). Compliance with this instruction was monitored on-line by the experimenter via an infrared video camera and was documented by electro-oculography (EOG; see below). No systematic changes in eye position were observed.

The experimental session comprised four blocks of equal duration, which were interrupted by short rest breaks (less than 10 minutes). In each block 480 sound stimuli were presented with a constant inter-stimulus interval of 1350 ms (1 stimulus per 1.5 s), thus resulting in total of 1920 sound stimuli per experimental session. The sound azimuth changed between stimulus presentations following a fixed quasi-random order, so that successive stimuli from the same loudspeaker were excluded. Sounds from all locations were presented with equal probability. The timing of the stimuli was controlled by custom-written software.

### Data recording and analysis

**Figure 1:**
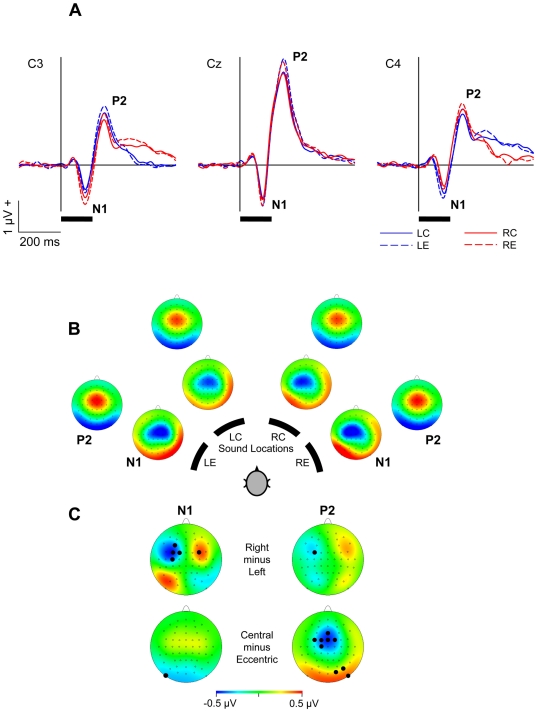
Auditory-evoked potentials. (**A**) Grand-average AEPs with N1 and P2 components at a left (C3), vertex (Cz), and right (C4) electrode position, plotted as a function of time relative to sound onset for left-eccentric (LE), left-central (LC), right-central (RC), and right-eccentric (RE) ranges of sound locations. Black horizontal bars indicate stimulus duration. (**B**) Topographies for the four ranges of sound locations (LE, LC, RE, RC) at the time of N1 and P2. (**C**) Difference topographies of N1 and P2, comparing right and left sound positions, and central and eccentric sound positions. Filled circles indicate electrodes with significant differences in amplitude values (significant *t*-values according to permutation tests, all *p*<0.05).

The continuous EEG was sampled at 500 Hz using 57 Ag/AgCl electrodes (referenced to a vertex electrode at FCz) and two cascaded NuAmps amplifiers (NeuroScan Labs, Sterling, VA). Electrode positions were based on the International 10-10 system (AF3, AF4, AF7, AF8, AFz, C1, C2, C3, C4, C5, C6, CP1, CP2, CP3, CP4, CP5, CP6, CPz, Cz, F3, F4, F7, F8, FC1, FC2, FC3, FC4, FC5, FC6, FCz, FP1, FP2, FPz, FT10, FT9, Fz, O1, O2, Oz, P1, P2, P3, P4, P7, P8, PO10, PO3, PO4, PO7, PO8, PO9, POz, Pz, T7, T8, TP7, TP8). Horizontal and vertical eye position was recorded by EOG using 4 additional electrodes positioned around both eyes. The ground electrode was placed at the center of the forehead, just above the nasion. Two additional electrodes were placed on the left and right mastoids. Electrode impedance was kept below 5 kΩ. The raw data were band-pass filtered off-line (cut-off frequencies 0.5 and 25 Hz; slope 48 dB/octave); low-pass filtering was used to remove residual high-frequency noise. The data were re-referenced to the average of 58 channels (56 EEG and 2 mastoid electrodes), and segmented into 1400-ms stimulus-locked epochs covering the period from −200 to 1200 ms relative to sound onset. As eye movements are inevitable in EEG experiments also when subjects are instructed to maintain fixation, data were corrected for ocular artifacts using the Gratton and Coles procedure [31]. Individual epochs exceeding a maximum-minimum difference of 200 µV were excluded from further analysis using the automatic artifact rejection implemented in the BrainVision Analyzer software (Version 1.05; Brain Products, Gilching, Germany). The remaining epochs were baseline corrected to a 200-ms pre-stimulus window and averaged for each listener and each sound condition. Peaks of the different event-related potential (ERP) components were defined as the maximum positivity or negativity within a particular latency window of specific waveforms (N1: 60–160 ms; P2: 160–260 ms after stimulus onset). The effects of hemispace and eccentricity of sound presentation on ERPs were tested by analyses of variance (ANOVAs) on amplitude and latency values of the C3, Cz, and C4 electrodes. For reasons of comprehensibility and in order to increase statistical power, data for four adjacent loudspeaker positions were collapsed, thus resulting in four data sets for analysis, each covering an azimuth arc of 40 degrees: *(1)* left eccentric (LE: −80°, −70°, −60°, −50°); *(2)* left central (LC: −40°, −30°, −20°, −10°); *(3)* right central (RC: 10°, 20°, 30°, 40°); *(4)* right eccentric (RE: 50°, 60°, 70°, 80°). Furthermore, we focussed on the two points in time with the largest root-mean-square power, that is, the N1 and P2 deflections (see Results; Fig. 1A).

Topographical differences of N1 and P2 to sound sources in the left (LC, LE) and right (RC, RE) hemispace, and to sound sources in central (LC, RC) and eccentric (LE, RE) positions were analyzed using the built-in permutation test (5000 permutations) of the EEGLAB toolbox [32]. The permutation test copes with multiple testing by permutation of the values of each participant across the experimental conditions (i.e., for the collection of tests performed for all electrodes; for reviews on this methodology and the technique of randomization statistics in neuroimaging, see, e.g., [33]).

### Cortical source localization

Source localization for the ERP components was carried out using sLORETA. LORETA [34] comprises a tomographic technique that gives a single solution to what is known as the inverse problem of location of cerebral sources [35]. sLORETA [20] is a new version of LORETA. The main difference is that sources are estimated on the basis of standardized current density allowing more precise source localization than the previous LORETA-method [20]. sLORETA calculates the standardized current density at each of 6239 voxels in the gray matter and the hippocampus of the Montreal Neurological Institute (MNI) brain template [36]. This calculation is based upon a linear weighted sum of the scalp electric potentials. sLORETA estimates the underlying sources under the assumption that neighboring voxels should have a maximally similar electrical activity (for details of this methodology, see [20]). sLORETA has been proven to achieve reliable localization of possible cerebral sources [37], [38]. sLORETA was performed within a 20-ms time window around the RMS peak of the average response (N1: 108 ms; P2: 208 ms). The voxel-based sLORETA-images of the AEPs (6239 voxels at a spatial resolution of 5 mm [20]) were compared with a 40-ms time period of silence immediately before stimulus onset, using the sLORETA-built-in voxelwise randomization tests. In addition, sLORETA was carried out for the N1 and P2 components of the AEP to reveal cortical regions, the activation of which significantly varied as a function of sound location. The voxels with significant differences (*p*<0.05) depending on sound locations were located in specific brain regions.

### ROI analyses

The ROI analyses were based on the general assumption that a genuine “spatial” cortical region may show changes of activation with variation of sound location. In detail, we hypothesized that the magnitude of voxel values (*t*-values) in “spatial” areas may change depending on two auditory spatial factors: *(1)* hemispace and *(2)* eccentricity of sound presentation. These factors were, thus, included in analyses of variance (ANOVAs) of voxel values.

Analyses of activations in specific regions of interest (ROIs) were conducted using the built-in ROI generator and extractor tools of the sLORETA software package. ROIs were equated to the sum of gray-matter voxels allocated to specific Brodmann areas (BAs) or structures as delivered by the sLORETA software (gray-matter volumes based on Talairach and Tournoux [39], [40]), and/or volumes defined by chosen coordinates. Overall, six ROIs were analyzed.

In an initial approach, we analyzed BA 41 (anterior transverse temporal gyrus or anterior Heschl's gyrus) bilaterally, with MNI coordinates (in mm) of centroids of *X* = −46.11, *Y* = −29.07, *Z* = 9.81 (left hemisphere; volume 3.38 cm^3^) and *X* = 46.61, *Y* = −28.57, *Z* = 10.00 (right hemisphere; volume 3.50 cm^3^). Referring to Hackett et al. [41], BA 41 closely corresponds with the core region of the human primary auditory cortex (A1). The main ROI analysis was focused on non-primary auditory areas and comprised five regions bilaterally. These regions were defined largely on the basis of the five brain regions of interest described by the meta-analysis of Arnott et al. [15]. As currently known, these regions correspond to the main “non-spatial” and/or “spatial” cortical regions processing auditory information beyond primary auditory cortex, as have been proposed in the dual-pathway model. The ROIs analyzed here were: *(1)* posterior superior temporal gyrus (pSTG), defined as the portion of the superior temporal gyrus with *Y*≤−35 mm; *(2)* IPL, defined as the total volume of BA 40; *(3)* SFS, defined by coordinates of *X* from ±20 to ±40 mm, *Y* from 0 to 20 mm, and *Z* from 45 to 70 mm; *(4)* aTL, defined as the portion of the temporal lobe with *Y*≥−10; and *(5)* IFG, defined as BAs 45 and 47 (all coordinates MNI). Centroids and sizes of these five ROIs are given in Table 1.

**Table 1:** Results of two-factor ANOVAs comparing either N1 or P2 responses to central (azimuth from ±10° to ±40°) and eccentric (from ±50° to ±80°) sound stimuli (factor “Eccentricity”) and stimuli on the left (from −80° to −10°) and right (from 10° to 80°; factor “Hemispace”) in 10 regions of interest (ROIs).

					Statistical values of the ANOVAs					
ROI	ROI Centroid Coordinates (MNI) [mm]			ROI Volume [cm^3^]	Hemispace (left vs. right stimuli)		Eccentricity (central vs. eccentric stimuli)		Hemispace×Eccentricity Interaction	
	*X*	*Y*	*Z*		N1	P2	N1	P2	N1	P2
Left pSTG	−53.14	−50.00	17.03	7.38	***F*** **_1,17_ = 17.28**	*F*_1,17_ = 1.63	*F*_1,17_ = 0.66	*F*_1,17_ = 0.00	*F*_1,17_ = 1.08	*F*_1,17_ = 1.49
					***p*** = 0.0007*	*p* = 0.22	*p* = 0.43	*p* = 0.96	*p* = 0.31	*p* = 0.24
Right pSTG	52.41	−48.30	15.98	7.00	***F*** **_1,17_ = 16.14**	*F*_1,17_ = 2.07	*F*_1,17_ = 0.10	*F*_1,17_ = 1.81	*F*_1,17_ = 0.02	*F*_1,17_ = 0.07
					***p*** = 0.0009*	*p* = 0.17	*p* = 0.76	*p* = 0.20	*p* = 0.90	*p* = 0.80
Left aTL	−46.53	3.74	−22.15	23.25	*F*_1,17_ = 0.19	*F*_1,17_ = 0.048	*F*_1,17_ = 0.30	*F*_1,17_ = 5.16	*F*_1,17_ = 2.29	*F*_1,17_ = 0.48
					*p* = 0.67	*p* = 0.83	*p* = 0.59	*p* = 0.036	*p* = 0.15	*p* = 0.50
Right aTL	47.75	3.85	−21.75	26.13	*F*_1,17_ = 0.39	*F*_1,17_ = 10.33	*F*_1,17_ = 2.11	*F*_1,17_ = 6.28	*F*_1,17_ = 10.39	*F*_1,17_ = 6.51
					*p* = 0.54	*p* = 0.0051	*p* = 0.16	*p* = 0.023	*p* = 0.0050	*p* = 0.021
Left IPL	−49.34	−42.83	40.24	23.63	***F*** **_1,17_ = 13.76**	*F*_1,17_ = 0.17	*F*_1,17_ = 6.83	*F*_1,17_ = 3.30	*F*_1,17_ = 0.11	*F*_1,17_ = 2.67
					***p*** = 0.0017*	*p* = 0.68	*p* = 0.018	*p* = 0.087	*p* = 0.74	*p* = 0.12
Right IPL	50.11	−43.00	40.58	22.50	***F*** **_1,17_ = 20.36**	*F*_1,17_ = 1.29	*F*_1,17_ = 4.58	*F*_1,17_ = 0.60	*F*_1,17_ = 9.55	*F*_1,17_ = 0.00
					***p*** = 0.0003*	*p* = 0.27	*p* = 0.047	*p* = 0.45	*p* = 0.0066	*p* 1.00
Left SFS	−28.68	9.82	55.26	7.13	*F*_1,17_ = 0.01	*F*_1,17_ = 2.46	*F*_1,17_ = 0.48	*F*_1,17_ = 1.42	*F*_1,17_ = 1.34	*F*_1,17_ = 1.37
					*p* = 0.93	*p* = 0.13	*p* = 0.50	*p* = 0.25	*p* = 0.26	*p* = 0.26
Right SFS	28.71	10.08	55.81	7.75	*F*_1,17_ = 0.11	*F*_1,17_ = 0.15	*F*_1,17_ = 0.17	*F*_1,17_ = 0.65	*F*_1,17_ = 0.64	*F*_1,17_ = 5.34
					*p* = 0.74	*p* = 0.70	*p* = 0.68	*p* = 0.43	*p* = 0.43	*p* = 0.034
Left IFG	−37.46	24.49	−7.35	17.00	*F*_1,17_ = 2.22	*F*_1,17_ = 3.82	*F*_1,17_ = 3.37	*F*_1,17_ = 1.77	*F*_1,17_ = 0.41	*F*_1,17_ = 0.77
					*p* = 0.15	*p* = 0.067	*p* = 0.083	*p* = 0.20	*p* = 0.53	*p* = 0.39
Right IFG	38.17	23.96	−7.45	17.38	*F*_1,17_ = 0.59	***F*** **_1,17_ = 13.26**	*F*_1,17_ = 0.063	*F*_1,17_ = 2.37	*F*_1,17_ = 5.72	*F*_1,17_ = 4.06
					*p* = 0.45	***p*** = 0.0020*	*p* = 0.81	*p* = 0.14	*p* = 0.029	*p* = 0.060

Asterisks and bold characters indicate effects that were statistically significant at the chosen alpha level (*α* = 0.0025).

Abbreviations: aTL, anterior temporal lobe; IFG, inferior frontal gyrus; IPL, inferior parietal lobule; pSTG, posterior superior temporal gyrus; SFS, superior frontal sulcus.

Referring to the “what/where” version of the dual-pathway model (see Introduction), we expected that the ANOVAs may reveal significant main effects and/or interactions in the putative “spatial” (IPL; SFS) and mixed “spatial/non-spatial” regions (A1; pSTG), rather than in the putative “non-spatial” regions (aTL; IFG).

## Results

### Auditory-evoked potentials

As shown in Fig. 1A, the onset of acoustic stimulation elicited a prominent vertex response. The AEP at the vertex position Cz was dominated by a negative deflection (N1) and a large second positive deflection (P2) at mean latencies of 108 ms and 208 ms respectively after sound onset (averaged across all sound locations). These components were also present at lateral electrode positions (C3 and C4; Fig. 1A). In addition, with contralateral stimulus locations a prominent positive deflection, most likely an offset P2 [42]–[44], was visible around 170 ms after sound offset; this was not further analyzed.

For the N1 deflection, an ANOVA with factors “Hemispace” (left vs. right sound locations), “Eccentricity” (central vs. eccentric sound locations), and “Hemisphere” (C3 vs. Cz vs. C4) indicated significant main effects of “Eccentricity” and “Hemisphere”, revealing greater N1 amplitudes to eccentric than central sounds (−1.16 vs. −1.00 µV; *F* [1], [17] = 7.22, *p* = 0.015), and on central than lateral electrode positions (C3: −0.92 µV; Cz: −1.38 µV; C4: −0.94 µV; *F* [2], [34] = 5.80, *p* = 0.01). There was no main effect of “Hemispace” (*F* [1], [17]<0.001, *p*>0.05), but a significant interaction of “Hemispace” and “Hemisphere” (*F* [2], [34] = 4.35, *p* = 0.037), suggesting that N1 amplitudes were greater above the hemisphere contralateral to the location of sound (Fig. 1A). To confirm this observation, amplitude values were averaged across central and eccentric locations, and values at contralateral locations (at C3 for right sound locations and a C4 for left sound locations) and ipsilateral locations (at C3 for left sound locations and a C4 for right sound locations) were submitted to a *t*-test, indicating significantly greater contralateral than ispilateral amplitudes (−1.04 vs. −0.82 µV; *t* [17] = 2.30, *p* = 0.034). An ANOVA on N1 latencies indicated a significant main effect of “Eccentricity” (*F* [1], [17] = 5.69, *p* = 0.029), with sightly shorter latencies to eccentric than central sounds (109.6 vs. 111.7 ms; *F* [1], [17] = 5.69, *p* = 0.029). In addition, there was a significant interaction of “Hemispace” and “Eccentricity” (*F* [1], [17] = 9.68, *p* = 0.006). Post-hoc *t*-tests on N1 latencies for each sound location (LC: 113.3 ms; LE: 108.7 ms; RC: 110.1 ms; RE: 110.6 ms) indicated longer latencies to left-central than to left-eccentric sounds (*t* [17] = 3.73, *p* = 0.001; Bonferroni-corrected values), while further differences did not reach statistical significance (all *p*>0.008).

For the P2 deflection, the ANOVA indicated significant main effects of “Eccentricity”, “Hemisphere”, and “Hemispace”, revealing greater P2 amplitudes to eccentric than central sounds (2.31 vs. 2.09 µV; *F* [1], [17] = 12.56, *p* = 0.002), to left than right sounds (2.25 vs. 2.15 µV; *F* [1], [17] = 5.14, *p* = 0.037), and on central than lateral electrode positions (C3: 1.65 µV; Cz: 3.24 µV; C4: 1.71 µV; *F* [2], [34] = 65.98, *p*<0.001). There were no significant interactions on P2 amplitudes, and no effects of sound condition on P2 latencies (all *p*>0.05).

Independently of sound position, the topographies of N1 and P2 showed negativity and positivity respectively over fronto-central cortex. In order to further investigate effects of “Hemispace” and “Eccentricity”, differences in topographies between left and right sound locations, and between central and eccentric locations were computed (Fig. 1C). The difference of right minus left locations revealed a left-hemispheric negativity and right-hemispheric positivity over fronto-central brain areas (in addition to a left-hemispheric, parieto-occipital positivity) for N1, and a slight left-hemispheric, fronto-central negativity for P2 (Fig. 1C). Accordingly, permutation tests indicated significant differences between left and right locations in topography of N1 (F3, FC3, FC1, C3, FC4), and P2 (FC3). The difference of central minus eccentric sound locations revealed a central negativity and a parieto-occipital positivity for P2, but only slight differences for N1 (Fig. 1B). Permutation tests indicated significant differences between central and eccentric locations in topography of N1 (PO9) and P2 (Fz, FC3, FC1, FCz, FC2, C1, O2, PO8, PO10; all *p*<0.05).

**Figure 2:**
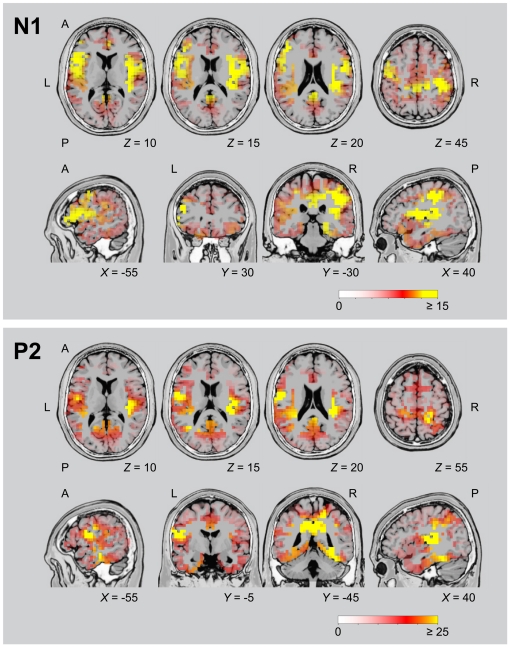
Peak activations of brain regions for all sound locations, as revealed by sLORETA analysis. Activations at the time of the N1 and P2 components of the responses to the sound onset were contrasted with a 40-ms prestimulus period of silence. Colour coding shows *t*-values, with statistically significant activations (*p*<0.05) at *t*≥4.1 for N1 and *t*≥3.6 for P2. Data from all subjects were projected onto a single anatomical image (T2 MNI-template “Colin 27” of sLORETA). Horizontal and coronal slices were positioned at MNI *Z* and *X* coordinates as given in the figure (A, anterior; L, left; P, posterior; R, right). Data are as given in Table 2.

Detailed analyses of the electric neural activity in specific brain regions at the time of the N1 and P2 deflections were conducted using sLORETA. For the N1 response, the contrast of spatial sound (data for all sound locations collapsed) versus silence (Fig. 2 and Table 2) revealed the most prominent activations in IPL (BA 40), postcentral gyrus (BAs 2, 3), precentral gyrus (BAs 4, 6), primary auditory cortex (BA 41), and insula (BA 13) of the right hemisphere, and in left middle frontal gyrus (BA 46). For the P2 response, the most prominent activations were obtained in left precentral gyrus (BAs 4, 6, 43), left postcentral gyrus (BA 3), right paracentral lobule (BA 5), right primary auditory cortex (BA 41), right insula (BA 13), and bilateral cingulate gyrus (BAs 23, 31).

**Table 2:** Locations of peak *t*-values for N1 and P2 responses to all sound positions vs. silence as revealed by sLORETA (all *p*<0.01).

			MNI Coordinates [mm]			
Deflection	Region	BA	*X*	*Y*	*Z*	*t*-Value
N1 Response						
	Right Hemisphere					
	Postcentral Gyrus	2	40	−30	30	19.21
	Inferior Parietal Lobule	40	45	−30	30	18.75
	Postcentral Gyrus	3	45	−25	40	17.76
	Postcentral Gyrus	40	40	−30	45	17.71
	Insula	13	40	−25	20	17.13
	Transverse Temporal Gyrus	41	40	−35	15	16.29
	Precentral Gyrus	6	50	−5	20	16.28
	Precentral Gyrus	4	40	−20	40	16.26
	Inferior Frontal Gyrus	44	50	0	20	15.97
	Left Hemisphere					
	Middle Frontal Gyrus	46	−45	30	25	16.17
P2 Response						
	Right Hemisphere					
	Paracentral Lobule	5	20	−45	50	32.14
	Insula	13	35	−20	15	31.03
	Transverse Temporal Gyrus	41	40	−25	10	28.27
	Parahippocampal Gyrus	19	30	−45	−5	28.26
	Precuneus	7	15	−45	50	28.04
	Superior Temporal Gyrus	41	35	−35	15	27.92
	Cingulate Gyrus	31	20	−35	40	27.88
	Fusiform Gyrus	37	35	−40	−10	27.78
	Cingulate Gyrus	23	5	−35	35	27.03
	Postcentral Gyrus	3	25	−35	50	26.85
	Left Hemisphere					
	Precentral Gyrus	4	−55	−5	15	32.30
	Precentral Gyrus	43	−50	−5	15	31.35
	Precentral Gyrus	6	−50	−5	20	29.56
	Cingulate Gyrus	31	−5	−40	40	29.35
	Postcentral Gyrus	43	−60	−10	20	29.33
	Precuneus	31	−15	−45	40	27.26
	Postcentral Gyrus	3	−30	−25	45	27.24
	Middle Temporal Gyrus	19	−35	−60	15	26.37
	Inferior Frontal Gyrus	44	−50	0	20	25.80
	Cingulate Gyrus	23	−5	−30	30	25.66

On the basis of a preceding fMRI study [22], we hypothesized that acoustically evoked activity in cortical areas associated with the analysis of spatial auditory cues would show co-variation with the sound location. Activations evoked by different sound directions were, thus, analyzed separately and statistical comparisons between these conditions were performed.

Two types of analysis were used to reveal brain areas that show significant differences in activation depending on the sector of sound directions. Firstly, we conducted ROI analyses (relying on *a priori* hypotheses on electrical activations) and secondly, we empirically analyzed contrasts between electrical responses to different sound locations.

### ROI analysis of primary auditory cortex

**Figure 3:**
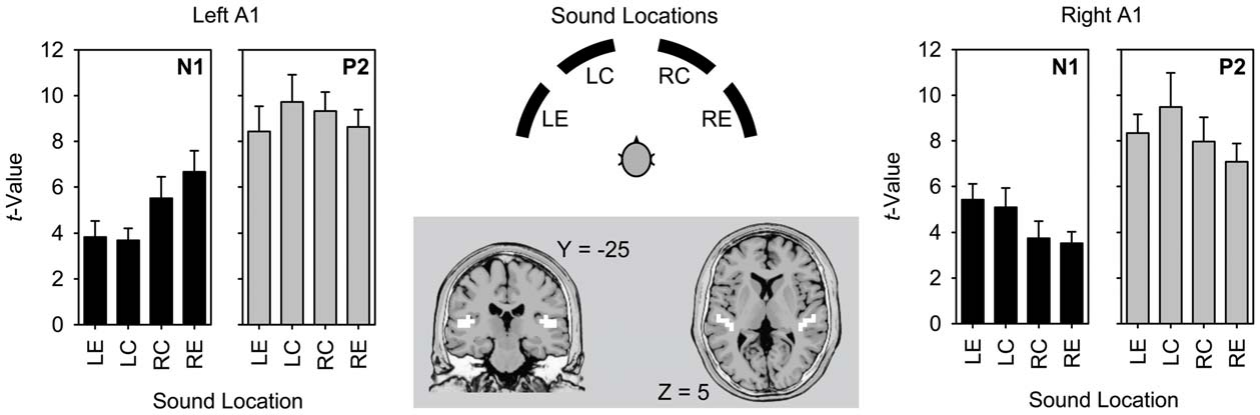
ROI-analysis of primary auditory cortex (BA 41) at the time of the N1 and P2. Data were collapsed for four adjacent loudspeaker positions, resulting in four data sets, each covering a range of 40 degrees (LE, left eccentric; LC, left central; RC, right central; RE, right eccentric; black arcs in the schematic view of the set-up). In the coronal and horizontal slices (MNI-template as in Fig. 2), voxels of the ROI are marked in white. The plots show *t*-values as a function of sound location (error bars, standard errors across subjects), resulting from contrasts of activations with a 40-ms prestimulus period of silence for the whole ROI.

The initial ROI analysis was focused on primary auditory cortex (Fig. 3). Using sLORETA software, for each voxel in left and right BA 41 the mean voxel values were computed for each of the four ranges of sound locations (left eccentric; left central; right central; right eccentric) and for each of the two deflections (N1, P2). Data for left and right BA 41 were normalized so that left and right hemispaces were assigned to ipsilateral and contralateral hemispaces with reference to the hemisphere of the ROI. All resulting data were entered into three-factor ANOVAs with “Hemisphere” (left, right), “Hemispace” (ipsilateral sound locations, contralateral sound locations), and “Eccentricity” (central sound locations, eccentric sound locations) as factors. For the N1 deflection, the ANOVA revealed a main effect of “Hemispace” (*F* [1], [17] = 41.58, *p*<0.0001, *η*_p_^2^ = 0.71), indicating generally greater activation by contralateral, than ipsilateral, sound. For the P2 deflection, the ANOVA revealed approaching significance for an effect of “Eccentricity” (*F* [1], [17] = 5.06, *p* = 0.038, *η*_p_^2^ = 0.23) at the chosen alpha level (Bonferroni-corrected for two ANOVAs: *α* = 0.05/2 = 0.025), suggesting a tendency of greater activation by central, than eccentric, sound. No additional main effects or interactions were found (all *F*≤1.65).

Subsequent *post-hoc* analyses were conducted for each hemisphere and each of the two deflections, using two-factor ANOVAs with “Hemispace” and “Eccentricity” as factors. These ANOVAs revealed main effects of “Hemispace” for the N1 in left BA 41 (*F* [1], [17] = 21.95, *p* = 0.0002, *η*_p_^2^ = 0.56) and right BA 41 (*F* [1], [17] = 8.73, *p* = 0.009, *η*_p_^2^ = 0.34), but no additional main effects or interactions (all *F*≤3.56; Fig. 3). Thus, taken together, both auditory cortices showed significantly higher activation when sounds were presented in contralateral, than ipsilateral, hemispaces at the time of the N1. This contralateral activation pattern had disappeared at the time of the P2.

### ROI analysis of non-primary auditory areas

**Figure 4:**
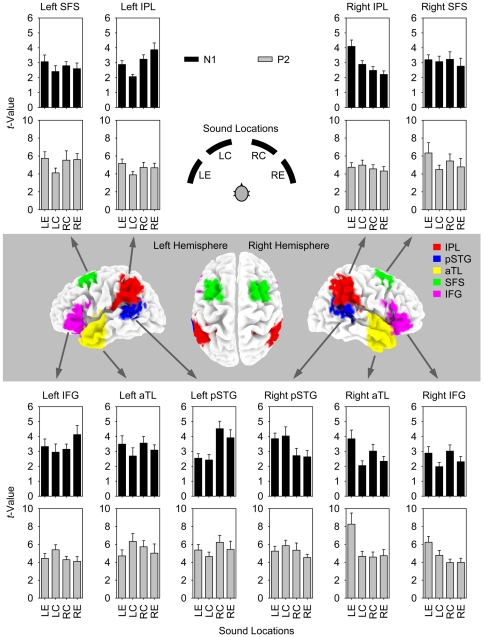
ROI-analysis of the five main cortical regions processing auditory information beyond primary auditory cortex. ROIs were posterior superior temporal gyrus (pSTG), inferior parietal lobule (IPL), superior frontal sulcus (SFS), anterior temporal lobe (aTL), and inferior frontal gyrus (IFG). Analyses were conducted for activation of the whole ROI at the time of the N1 and P2 components of the responses to different sound locations (as in Fig. 3). ROIs are mapped onto the standard 3-D MNI brain template “Colin” of sLORETA. The plots show *t*-values as a function of sound location (error bars, standard errors across subjects; N1, black bars; P2 gray bars), resulting from contrasts of activations with a 40-ms prestimulus period of silence for the whole ROI. Data are as given in Table 1.

The main ROI analysis was based on the meta-analysis of Arnott et al. [15], who reviewed “spatial” and “non-spatial” auditory functional imaging studies in order to determine the reliability of the dual-pathway model in humans. The ROIs were chosen according to the five “spatial” and “non-spatial” brain regions analyzed by these authors: *(1)* posterior superior temporal gyrus (pSTG); *(2)* inferior parietal lobule (IPL); *(3)* superior frontal sulcus (SFS); *(4)* anterior temporal lobe (aTL); and *(5)* inferior frontal gyrus (IFG), all bilaterally (Fig. 4). As with the ROI analysis of auditory cortex, for each voxel in each ROI the mean voxel values were computed for left eccentric, left central, right central, and right eccentric locations and for each of two deflections (N1, P2). All resulting data for each deflection and each of the ten ROIs were entered into a two-factor ANOVA with “Hemispace” and “Eccentricity” as factors (Bonferroni-corrected for 20 ANOVAs: *α* = 0.05/20 = 0.0025). The results of these ANOVAs are reported in Table 1 and the corresponding plots of mean voxel values as a function of sound location for each ROI are shown in Fig. 4. Significant main effects of “Hemispace” (*F* [1], [17]≥13.26, *p*<0.002) were obtained for left and right pSTG and left and right IPL at N1, and for right IFG at P2, with the typical contralaterality pattern observed in each case. Main effects of “Eccentricity” were not statistically significant at the chosen alpha level, even though there was some approaching significance (*p*-values from 0.018 to 0.047) for left and right IPL at N1, as well as for left and right aTL at P2. The “Hemispace”×“Eccentricity” interaction very closely approached the chosen level of significance at N1 in right aTL (*F* [1], [17] = 10.39, *p* = 0.0050, *η*_p_^2^≥0.38) and in right IPL (*F* [1], [17] = 9.55, *p* = 0.0066, *η*_p_^2^≥0.36), and, to a lesser degree, in right IPL and right IFG at N1, and in right aTL and right SFS at P2 (*p*-values from 0.021 to 0.034). This tendency suggested an asymmetrical pattern of eccentricity sensitivity, with stronger activation with eccentric, than central, sound in left hemispace and stronger activation with central, than eccentric, sound in right hemispace (see, e.g., the plot for right aTL/N1 in Fig. 4).

Taken together, significant co-variation of electrical activity with sound location was found in bilateral pSTG and bilateral IPL at N1, and in the right IFG at P2. This generally provides evidence of a shift of spatially sensitive neural activity, within the 100-ms interval between N1 and P2, from the temporo-parietal regions of the dorsal auditory pathway to inferior frontal cortex that has been assigned to the ventral auditory pathway.

### Contrasts between sound locations

**Figure 5 pone-0025146-g005:**
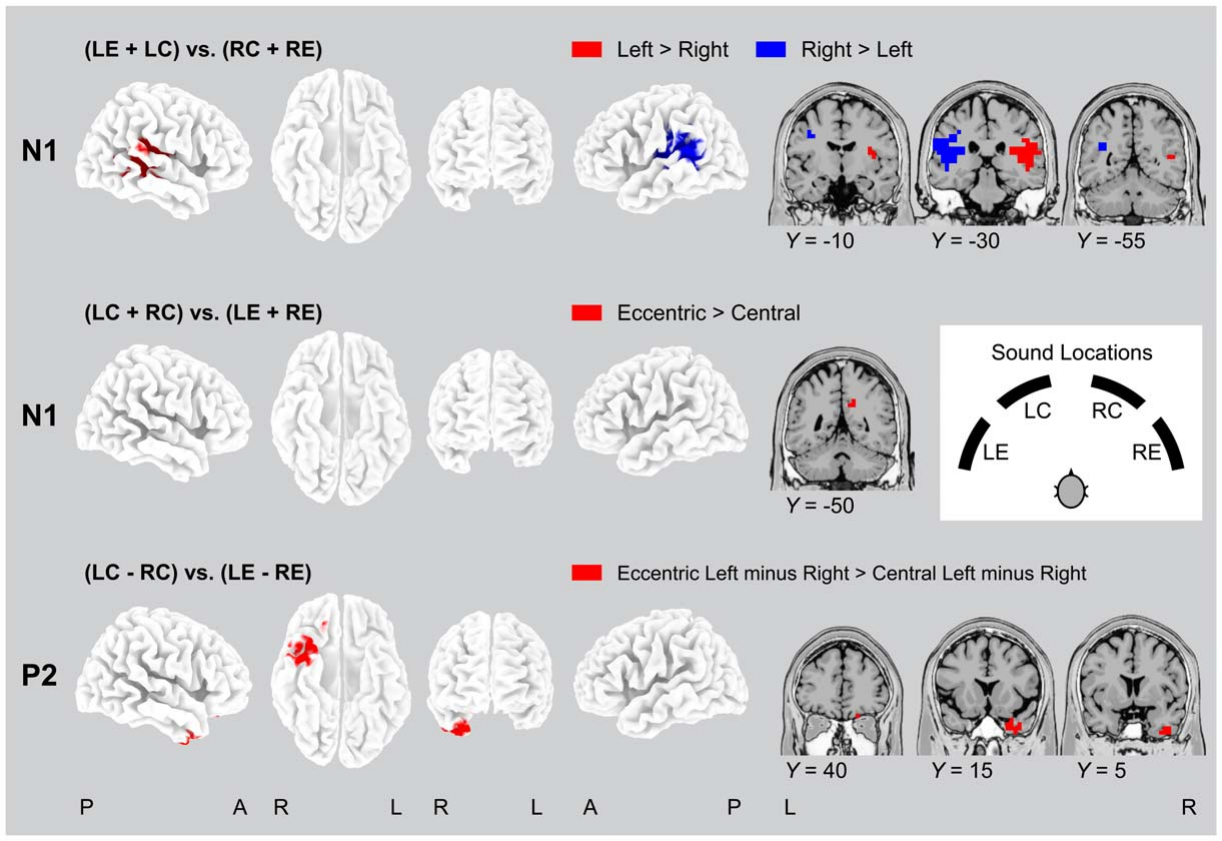
Activations of cortical regions as revealed by different contrasts. The contrasts of left vs. right [(LE+LC) vs. (RC+RE)] and central vs. eccentric sound locations [(LC+RC) vs. (LE+RE)] indicated significant activations exclusively at the N1 component of the responses to different sound locations. The more complex interaction of the difference of left and right central locations vs. the difference of left and right eccentric locations [(LC−RC) vs. (LE−RE)] was significant only at the P2. Contrasts are mapped onto a standard 3-D brain template and coronal slices (MNI-templates as in Figs. 2, 4). Data are as given in Table 3. LE, left eccentric; LC, left central; RC, right central; RE, right eccentric. No significant activations were found for the left vs. right and central vs. eccentric sound locations at the P2, and for the interaction at the N1.

**Figure 6:**
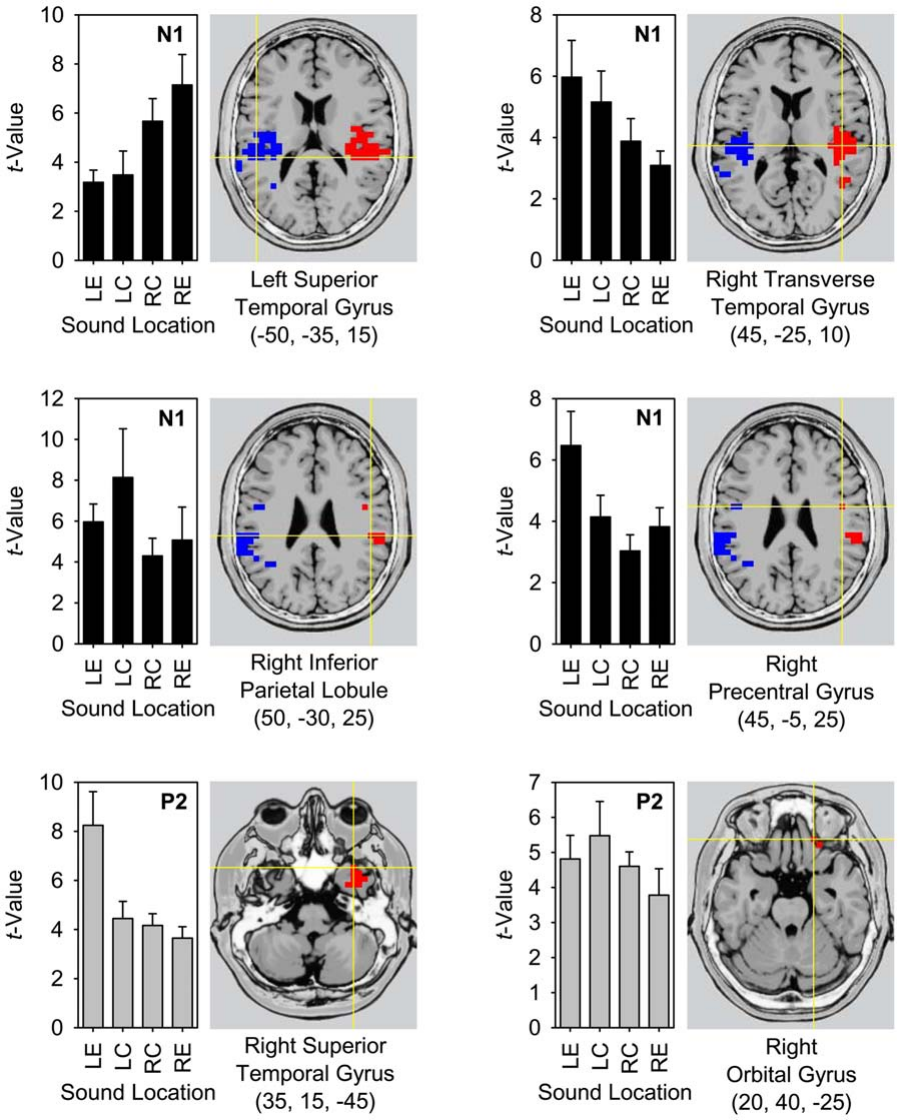
Significant variation of activation as a function of location for six examplary areas, as revealed by different contrasts either at the time of the N1 (black bars) or at the time of the P2 (gray bars). For the voxel with maximum activation in each of the areas (coordinates taken from Table 3), the plots show the mean *t*-values as a function of sound location (error bars, standard errors across subjects), resulting from contrasts of activations with a 40-ms prestimulus period of silence (as in Figs. 3, 4). Note that these *t*-values are based on statistical comparisons of the estimated current densities at a specific sound location versus baseline, whereas the images are based on comparisons between estimated current densities for different sound locations. Coordinates given in brackets indicate *X*, *Y*, *Z* MNI coordinates of the maximum activation, as are shown in horizontal slices. Areas are as in Table 3 and Fig. 5.

In the second main analysis, electrical activations evoked by different sound locations (left eccentric, left central, right central, right eccentric) were analyzed separately, and contrasts between these conditions were computed using the statistical non-parametric mapping tools of the sLORETA software package (Fig. 5; Table 3). The contrast of left versus right sound locations for the N1 deflection revealed prominent bilateral activations in primary auditory cortex (BAs 41, 42), pSTG (BA 22), IPL (BA 40), and insula (BA 13), as well as unilateral activations in right precentral gyrus (BA 6) and in the left temporo-occipital region (BA 19). This contrast showed a clear-cut contralaterality pattern, with opposing signs of voxel values in left and right hemispheres (see Figs. 5, 6; Table 3). The contrast of central versus eccentric sound locations only revealed one right-hemisphere cluster of activated voxels for the N1 response, which was located in the border area of precuneus and cingulate gyrus (BA 31). No significant contrasts of left versus right sound locations or central vs. eccentric sound locations were found for the P2 response.

**Table 3:** Processing of left, right, central, and eccentric sound locations (all *p*<0.05).

			MNI Coordinates [mm]			
Contrast and Deflection	Region	BA	*X*	*Y*	*Z*	*t*-Value
*Left vs. Right Sound Locations*						
N1 Response						
	Temporal					
	Right Superior Temporal Gyrus	41	45	−30	15	6.96
	Right Transverse Temporal Gyrus	41	45	−25	10	6.96
	Right Superior Temporal Gyrus	22	50	−30	5	6.34
	Right Middle Temporal Gyrus	22	50	−35	5	6.32
	Right Superior Temporal Gyrus	42	55	−30	15	5.74
	Right Superior Temporal Gyrus	39	45	−55	10	4.99
	Right Middle Temporal Gyrus	21	55	−45	5	4.54
	Right Inferior Temporal Gyrus	20	50	−50	−10	4.22
	Right Middle Temporal Gyrus	39	45	−60	10	4.21
	Left Superior Temporal Gyrus	41	−50	−35	15	−8.00
	Left Superior Temporal Gyrus	42	−55	−35	15	−7.20
	Left Transverse Temporal Gyrus	41	−45	−30	10	−6.98
	Left Superior Temporal Gyrus	22	−60	−40	20	−6.56
	Left Middle Temporal Gyrus	22	−50	−35	5	−6.42
	Left Middle Temporal Gyrus	21	−55	−45	5	−5.27
	Left Superior Temporal Gyrus	39	−35	−55	25	−5.20
	Left Supramarginal Gyrus	40	−50	−50	20	−5.15
	Left Middle Temporal Gyrus	39	−35	−60	20	−4.28
	Parietal					
	Right Postcentral Gyrus	40	50	−25	15	6.40
	Right Inferior Parietal Lobule	40	50	−30	25	6.01
	Right Precuneus	31	20	−45	35	4.35
	Right Postcentral Gyrus	43	50	−15	15	4.25
	Left Postcentral Gyrus	40	−55	−30	20	−6.39
	Left Inferior Parietal Lobule	40	−50	−30	25	−6.28
	Left Postcentral Gyrus	2	−40	−30	30	−5.84
	Left Postcentral Gyrus	43	−50	−15	15	−4.24
	Frontal					
	Right Precentral Gyrus	6	45	−5	25	4.39
	Occipital					
	Left Middle Temporal Gyrus	19	−35	−60	15	−4.62
	Insula					
	Right Insula	13	45	−30	20	6.89
	Left Insula	13	−50	−35	20	−7.52
*Central vs. Eccentric Sound Locations*						
N1 Response						
	Parietal					
	Right Precuneus	31	15	−50	35	−4.38
	Right Cingulate Gyrus	31	15	−50	30	−4.31
*Left Central minus Right Central vs. Left Eccentric minus Right Eccentric*						
P2 Response						
	Temporal					
	Right Superior Temporal Gyrus	38	35	15	−45	−5.01
	Right Middle Temporal Gyrus	38	40	10	−45	−4.91
	Right Middle Temporal Gyrus	21	40	10	−40	−4.75
	Right Inferior Temporal Gyrus	20	35	0	−45	−4.55
	Frontal					
	Right Orbital Gyrus	47	20	40	−25	−4.42
	Right Inferior Frontal Gyrus	11	25	35	−25	−4.35

As the ROI analyses described above (cf. plots in Fig. 4) as well as previous fMRI findings [22] indicated complex interactions between hemispace and eccentricity sensitivity, we finally computed the contrast of left central minus right central sound locations versus left eccentric minus right eccentric sound locations (Figs. 5, 6; Table 3). No such contrasts approached significance for the N1 response. However, for the P2 deflection, contrasts revealed activations in the most anterior aspects (all voxels at MNI *Y*≥0 mm) of the right inferotemporal cortex, involving superior temporal gyrus, middle temporal gyrus, and inferior temporal gyrus (BAs 20, 21, 38), and in right orbital frontal cortex, namely in orbital gyrus and inferior frontal gyrus (BAs 11, 47). This analysis suggested that at the time of the P2 the relationship between cortical activation and sound location had become more complex (i.e., non-monotonic) than the simple contralaterality pattern found at the time of the N1.

Figure 6 shows examplary plots of the variation of activation (*t*-values) of single voxels (coordinates as in Table 3) as a function of stimulus position. The general pattern of more intense activation with contralateral than ipsilateral sound obviously remained unchanged across areas and deflections. However, an increase of activation with more contralateral position was observed only in posterior temporal cortex and only at the time of the N1. In areas beyond this region and particularly at the time of the P2, plots rather showed maxima either at contralateral-central or at contralateral-eccentric stimulus locations, thus suggesting a transition from monotonic to non-monotonic azimuth functions.

## Discussion

As the main result, we found a clear-cut double dissociation with respect to the locations and the points in time of auditory spatial processing in the human cortex: while posterodorsal processing was obtained at the time of the N1, processing was displaced to anteroventral areas at the time of the P2. Moreover, the analysis of contrasts between sound locations showed some hints that the posterodorsal N1 activation, in particular in the posterior temporal region, could be compatible with a population rate coding of sound azimuth (i.e., increasing activation with increasing contralaterality of stimuli), whereas a more complex integration of information on stimulus hemispace and eccentricity may take place at the anteroventral P2 activation.

### The “where” of auditory spatial processing

At the time of the N1, variation of electrical cortical activity depending on sound location, as revealed by sLORETA, was mainly found in the region of the TPO junction (the junction area between the temporal, parietal and occipital lobes), including the core region of the primary auditory cortex (BA 41), the posterior aspects of the superior temporal and middle temporal gyri, insula, as well as parts of precentral and postcentral gyri and IPL. In addition, the contrasts between sound locations (though not the ROI analysis) revealed activation in dorsofrontal cortex (BA 6), directly adjacent to the SFS region. Thus, our findings almost perfectly correspond to earlier work by demonstrating the involvement of the posterodorsal pathway (from primary auditory cortex via pSTG and IPL to dorsofrontal cortex) in spatial auditory functions (see literature cited below). In this respect, it is remarkable that electrotomography revealed virtually identical locations of cortical activation as imaging techniques based on haemodynamic signals (fMRI: e.g., [10], [11], [22], [23], [45], [46]; PET: e.g., [9], [47]–[50]). Similarly, these findings are fully compatible with EEG (e.g., [51]–[56]) and magnetoencephalography (MEG) source analyses (e.g., [26], [57]–[60]). Also, the posterior-temporal/parietal region has been shown to play an important role in sound localization by transcranial magnetic stimulation (TMS) studies [61]–[63], studies with patients suffering from cortical lesions [64]–[70], and single-neuron recordings in the monkey [71], [72].

At the time of the P2, sLORETA revealed spatially sensitive activity in the anteroventral pathway, namely in aTL (in contrasts between sound locations, but not ROI analysis) and IFG (in both contrast and ROI analyses). This result appears to be in opposition to the majority of previous imaging studies on auditory spatial processing in cortex, that failed to reveal anteroventral activations (see meta-analysis of Arnott et al. [15]). However, even though there are only a few imaging studies that have provided evidence of auditory spatial processing in the anteroventral pathway in addition to the posterodorsal region (e.g., [21], [22], [73], [74]), this minority of positive results cannot be disregarded. In particular, a recent study using fMRI in combination with a similar experimental paradigm as employed here [22] obtained covariation of activity with the sound location in both aTL (BA 21, 38) and IFG (BA 47), as was found here at the time of the P2. Interestingly, in substantial alignment with the contrasts in the present study, these anteroventral activations became manifest only with the interaction of the factors “hemispace” and “eccentricity” of sound, whereas posterodorsal activations were found primarily with the contrast of left versus right sound locations. Thus, if one considers the lack of temporal resolution with fMRI, the present electrotomography results confirmed the main findings of the fMRI study [22]. Finally, an involvement of aTL in spatial hearing has been also demonstrated by studies that showed impairment of sound localization after circumscribed lesions of this region [75]–[77].

On the basis of several imaging studies (e.g., [12], [53]; for review, see [15]), it is generally accepted that the anteroventral pathway is concerned with spectrotemporal analysis in order to enable the identification of the source of the sound (see, however, [78]). Given the functional duality of auditory spectral analysis – that is, the concurrent extraction of information based on location (due to the spectrotemporal distortions caused by body, head, and pinnae) and spectral characteristics of a sound source – it has been hypothesized that regions specialized in spectral analysis, namely those in the anteroventral auditory stream, may be shared by object-feature processing and spatial processing of realistic sound sources [22] (cf. also [21], [50]). This view is also supported by perceptual phenomena, e.g., the long-known auditory illusion that variation of the pitch of a sound source can result in variation of its apparent spatial location, particularly in elevation [79], but also in azimuth [80]. Notable is that the assumption of shared networks for sound identification and spatial analysis perfectly fits studies in the monkey, which reported similar spatial and non-spatial sensitivities of neurons in ventral prefrontal and lateral intraparietal cortex [81], [82]. In a more general context, this conclusion may also be in alignment with recent neuropsychological findings suggesting that in human brain spatial processing is strongly linked with functions of pitch perception [83].

### Methodological considerations

The question arises of why the overwhelming majority of previous imaging studies on auditory spatial processing had, unlike the present study, failed to reveal anteroventral areas. At the first glance, one might assume that differences in the imaging techniques used could have played any role, namely whether imaging was based on electrical (present study) or haemodynamic responses (fMRI/PET studies). However, this possibility seems less likely. As already mentioned above, the present results were consistent with those of the preceding fMRI study [22] with respect to the locations of activations. Furthermore, activation in PET studies of Griffiths and Green [73] and Zatorre et al. (Experiment 3/L5) [21] were located in the anteroventral region. Interesting in this respect is that all these investigations including the present one (although employing quite different active or passive psychophysical paradigms) critically differed in two methodological points from studies that failed to find anteroventral foci of activation.

Firstly, the present study, as well as those mentioned above [21], [22], [73], used acoustic stimuli that most effectively took into account the complete set of localization cues available to the auditory system under natural conditions. This was implemented by stimulation in the free sound field under well-defined anechoic conditions (present study), quasi free-field sound presentation within the PET scanner [21], presentation of individual binaural recordings [73] or individualized head-related transfer function (HRTF) based stimuli [22] via headphones. This issue is essential for the interpretation of the results of these studies. Under the realistic conditions of a complex free-field sound source, auditory localization is based not only on analysis of interaural differences in sound pressure level (ILDs) or time of arrival (ITDs), but also on spectral localization cues. These latter cues are distortions in the overall spectral shape of the incoming sound and differences in the frequency spectra between the ears, produced by the listener's body, head and pinnae. Their existence is crucial for emergence of a natural sound image in external space [80], [84]–[88]. The importance of spectral localization cues for investigations on cortical processing of auditory spatial information has been recently demonstrated in an EEG study on auditory motion processing [89]. In particular, this study supported the view that natural-like stimulation in the free sound field may yield substantially more reliable data on auditory spatial processing than non-individualized (artificial-head) HRTF-based stimuli or artificial stimuli generated by ILDs or ITDs. To our knowledge, imaging studies that did not use free-field sound stimuli or individualized natural-like reproduction of all localization cues via headphones failed to find anteroventral activation depending on sound location.

The second methodological point to be emphasized is that in the present study and in other imaging studies that have described anteroventral activation [21], [22], [73], contrasts were computed between different conditions of spatial acoustic stimulation. Unlike that, most other studies have analyzed single contrasts between an active task of sound localization (often involving a motor response) and either silence, passive listening or “non-spatial” tasks such as pitch discrimination (for review, see [15]). Those imaging results may involve contamination with unspecific factors that are quite difficult to control, and results may thus be generally less reliable with respect to the identification of “spatial” auditory brain areas (for a detailed discussion, see [22]). In particular, analyses contrasting “spatial vs. non-spatial” tasks (both involving spectrotemporal processing) may not reveal any activations related to the neural analysis of spectrotemporal localization cues. Thus, it seems rather likely that those “spatial vs. non-spatial” contrasts actually reflected the contrast between *(1)* processing of binaural spatial (ITD/ILD) cues and *(2)* both the spatial and non-spatial aspects of spectrotemporal processing.

A further methodological point to be mentioned is that there might have been cross-talk among the current source estimates, thus resulting in incorrect localization of activation by sLORETA, particularly at neighbouring locations [90]–[92]. It was shown that using sLORETA, simultaneously active sources can only be separated if their fields are distinct enough and of similar strength [93]. We cannot completely exclude that this problem was relevant with respect to the spatial separation of adjacent areas of activations, namely separation between aTL and IFG, and between pSTG and IPL. However, the Euclidean distances between ROI centroids (Table 1) and the Euclidean distances between coordinates of peak activations, revealed by contrasts in different ROIs (Table 3), were larger than 20 mm, which is beyond the limits given by the low spatial resolution of cortical current density imaging techniques such as sLORETA [93], [94].

### Relation of “when” and “where” of auditory spatial processing

In sum, our finding of spatially sensitive activations in both pathways beyond A1 indicated that processing and relaying of the auditory spatial information takes place in the entire dual-pathway network of the auditory cortical system, encompassing all areas known to be concerned with the analysis of sound features in general. However, there was a striking double dissociation regarding the chronology of processing and regions of activation. At the time of the N1 (about 100 ms after stimulus onset), processing involved A1 and the posterodorsal pathway but not anteroventral areas. One hundred milliseconds later (at the time of the P2) these activations were below the level of significance and spatial processing was present in areas of the anteroventral pathway.

How can this clear-cut double dissociation be explained? It is well established from several studies that the N1 is related to posterior activations and the P2 has a more anterior effect in temporal lobe (e.g., [13], [95]–[101]). While the N1 is known to be sensitive to onset parameters of sound stimuli [101], the P2 has been suggested to reflect the neural analysis of spectral complexity of acoustic stimuli [102]. It seems plausible that these principles, though originally proposed for non-spatial analysis, can be applied to spatial functions as well. As was argued above, we assume that activation of the posterodorsal pathway was specifically associated with the analysis of ITD/ILD localization cues while the anteroventral pathway may be specialized in analysis of spectral localization cues. It is clear that analyses of interaural differences in onset time, phase and level can be performed within only a few cycles of the waveform of the sound, that is, within a short time window after sound onset. In contrast, the more subtle analysis of spectral localization cues necessarily demands a sufficient, much longer time interval due to the periodicity processing (for psychophysical evidence, see [103]). Thereby, the initial analyses of both the ITD/ILD cues and the spectral cues (usable for localization) may start in parallel already at the level of the brainstem, but in separate structures: ILDs and ITDs are initially processed in the superior olivary complex (lateral superior olive for ILDs, medial superior olive for ITDs; for review, see [104]) and spectral cues in the dorsal cochlear nucleus [105]–[108]. There is some indication that initial convergence of the different binaural cues may occur in the central nucleus of the inferior colliculus [109]–[112]. However, other evidence suggests that representations of ITDs and ILDs remain separate even at the level of the auditory cortex [52], [113], [114]. Evidence for a double dissociation in cortical processing of auditory spatial information has also been demonstrated in a recent EEG study on motion perception [115], indicating that the early and late motion-specific ERPs reflect different phases in motion processing. While the change-N1 appeared to be sensitive to the hemifield within which a sound moves (thus coding the direction-independent onset position), the change-P2 rather reflected the motion direction (thus coding the more complex location-independent direction of spatial change).

Thus, on this basis, one may conclude that ITD/ILD information may be earlier available for further processing in cortex (at the time of the N1) than spectral information (at the time of the P2). This view has been experimentally substantiated in an MEG study [58]. The comparison of the reactivity of the human auditory cortex to sound azimuth (mainly relying on ITD/ILD cues) and elevation (mainly relying on spectral cues) indicated that spectral localization cues were processed in the auditory cortex about 100 ms later than the ITD/ILD cues. This time difference is identical with the period between N1 and P2 peaks in the present study. Furthermore, Tiitinen et al. [116] using MEG with passive listening, found that the right-hemispheric P2m, unlike the earlier P1m and N1m components of the neuromagnetic response, was more sensitive to natural-like 3D sounds than to ITD stimuli, thus reflecting the degree of “spatiality” of sound. These authors consequently concluded that the right hemisphere is specialized in the processing of natural-like spatial information, including the spectral localization cues, while ITDs are processed equally in both hemispheres. Interestingly, the proposed time difference between processing of ITD/ILD cues and spectral cues perfectly matches the recent finding of Altmann et al. [53], who found out that changes in sound location were processed faster than changes in sound pattern by about 100 ms. If combined with the result of the same authors that pattern changes were processed more anteriorly in superior temporal lobe and location changes more posteriorly, this relation may support the hypothesis of a sharing of the anteroventral auditory network by object-feature processing and spatial processing much more.

Moreover, it might be that the aTL is the locus where auditory ITD/ILD information is integrated with the spectral (spatial and object-feature) information at the time of the P2 (cf. also [13]). For the visual modality it has been proposed that the visual input is projected very early and rapidly via the dorsal visual pathway from visual cortex to orbital cortex/IFG, in parallel to the relatively slower processing along the ventral pathway in temporal cortex, and that feedback connections from orbitofrontal cortex/IFG to aTL via the uncinate fasciculus initiate top-down facilitation of object recognition [117], [118]. The present results could be compatible with the existence of a related feedback mechanism in the auditory modality insofar as the early posterodorsal processing of spatial information could trigger the slower and more complex spectral processing in aTL via IFG and uncinate fasciculus, thus linking the complete set of spatial and non-spatial components of the auditory information at the time of the P2. On the basis of the present data, this possibility is, however, still a matter of speculation. Our results confirmed not only the general view of right hemisphere superiority or dominance for the processing of sound location, as has been suggested in several neuroimaging studies [45], [57], [119]. Rather, as shown in Fig. 5, they indicated that this bilateral asymmetry pattern was largely confined to activations in anteroventral pathway and to the time of the P2. At the time of the N1, left and right activation foci in pSTG and IPL were roughly similar, without any obvious advantage of one hemisphere. In accordance with this latter finding, studies that investigated acallosal or callosotomy subjects suggested that transfer of auditory spatial information via the corpus callosum plays a significant role in sound localization [120], [121]. Additionally, investigations with brain-damaged subjects indicated that total inability of sound localization or lateralization can occur in individual patients with left-hemispheric lesions and those with right-hemispheric lesions, but severe deficits are usually observed more frequently in the latter group [64], [76], [121], [122].

### Implications for auditory space coding

The contrasts between sound locations suggested that different levels of complexity of spatial coding may exist in different cortical areas and at different points in time. A relatively simple type of coding seemed to take place first, primarily in posterior superior temporal lobe. Here, electrical activation increased roughly monotonically with variation of sound position from ipsilateral to contralateral locations, as evidenced by the contrast of left vs. right sound locations (see Figs. 2, 3, 5, 6). This may be in alignment with the (even though weakly pronounced) contralaterality in the auditory cortical system, as was known from previous studies [123]. An isolated coding of sound eccentricity (i.e., central vs. eccentric locations) obviously did not play a decisive role, as only marginal activation in the cingulate region, but not in the main auditory cortical pathways, was found for co-variation with eccentricity at the time of the N1 (cf. Fig. 5). These results were compatible with the proposal of a population rate code for auditory space in human cortex. That is, auditory space would be represented in that the neural populations in left posterodorsal pathway are preferentially activated by sound sources to the right and the neural populations in right posterodorsal pathway by those to the left of the listener. The location of a sound source would be then encoded in the relative level of activity in these two groups of neurons [124]. However, primarily (but not exclusively) at the time of the P2 and in anteroventral pathway, the relation of activation and sound location appeared to become more complex (see Tables 1, 3; Figs. 4–6). Whether this latter finding reflected a fundamentally changed type of coding or the simultaneous existence of different types of coding in the same population remained, however, unclear and has to be investigated further.

### Conclusion

The clear-cut double dissociation found with respect to the cortical locations and the points in time of auditory spatial processing indicated early processing in primary auditory cortex and posterodorsal auditory cortical pathway, whereas about 100 ms later spatial processing was displaced to anteroventral areas. Thus, both auditory pathways are apparently involved in spatial analysis, but at different points in time. In accordance with the conclusions of several earlier studies [21], [22], [55], [81], [82], our findings suggest that there could be a functional dissociation of both of these pathways insofar as they process and relay different aspects of the auditory spatial information. It seems possible that the ITD/ILD cues are preferentially processed in posterodorsal areas while in the anteroventral areas spatial and non-spatial functions of spectral analysis could be shared. In general, this hypothesis is in alignment with the currently discussed revision of the auditory dual-pathway model that assumes that – analogous to the visual cortical streams [18], [19] – spatial and non-spatial auditory information is processed within both pathways, with the posterodorsal pathway being concerned with the preparation of action in response to auditory stimuli and the anteroventral pathway assigned to perceptual auditory functions (cf., e.g., [16], [17]).
